# Spatial Distribution of a Porphyrin-Based Photosensitizer Reveals Mechanism of Photodynamic Inactivation of *Candida albicans*

**DOI:** 10.3389/fmed.2021.641244

**Published:** 2021-07-19

**Authors:** Thomas Voit, Fabian Cieplik, Johannes Regensburger, Karl-Anton Hiller, Anita Gollmer, Wolfgang Buchalla, Tim Maisch

**Affiliations:** ^1^Department of Dermatology, University Hospital Regensburg, Regensburg, Germany; ^2^Department of Conservative Dentistry and Periodontology, University Hospital Regensburg, Regensburg, Germany; ^3^Private Practice, Kaufbeuren, Germany

**Keywords:** antifungal, photodynamic, porphyrin, *Candida albicans*, microscopy

## Abstract

The antimicrobial photodynamic therapy (aPDT) is a promising approach for the control of microbial and especially fungal infections such as mucosal mycosis. TMPyP [5,10,15, 20-tetrakis(1-methylpyridinium-4-yl)-porphyrin tetra p-toluenesulfonate] is an effective photosensitizer (PS) that is commonly used in aPDT. The aim of this study was to examine the localization of TMPyP in *Candida albicans* before and after irradiation with visible light to get information about the cellular mechanism of antifungal action of the photodynamic process using this PS. Immediately after incubation of *C. albicans* with TMPyP, fluorescence microscopy revealed an accumulation of the PS in the cell envelope. After irradiation with blue light the complete cell showed red fluorescence, which indicates, that aPDT is leading to a damage in the cell wall with following influx of PS into the cytosol. Incubation of *C. albicans* with Wheat Germ Agglutinin (WGA) could confirm the cell wall as primary binding site of TMPyP. The finding that the porphyrin accumulates in the fungal cell wall and does not enter the interior of the cell before irradiation makes it unlikely that resistances can emerge upon aPDT. The results of this study may help in further development and modification of PS in order to increase efficacy against fungal infections such as those caused by *C. albicans*.

## Introduction

*Candida albicans* is known as the most common pathogenic fungus within the human body causing a variety of superficial and invasive fungal infections (1). This fungus can be found throughout the whole gastrointestinal tract from the oral cavity to the rectum (2). Especially patients wearing dentures suffer from candidiasis of the oral mucosa, which is often associated with pain (3, 4).

*C. albicans* has a complex cell wall which protects the cell and plays an important role in the interaction between fungus and host (2). The fungal cell wall consists of an outer and an inner layer. The inner layer of the cell wall contains chitin and β-1,3-glucan, whereas N- and O- linked mannose polymers, that are covalently bound to proteins, are components of the outer layer. Both layers arelinked by β-1,6-glucan (2, 5). The cell wall structure of *C. albicans* could be an obvious reason, why the fungus usually exhibits higher tolerance toward antimicrobial therapy as compared to most bacteria (6, 7). Common antimycotic agents used for control of *C. albicans* infections are polyenes, azoles and echinocandins (8, 9). Particularly for azoles like fluconazole, many authors describe resistant *C. albicans* strains, which issues a challenge to the whole healthcare system (10, 11). Therefore, it is reasonable to develop novel antimicrobial strategies in the therapy of fungal infections. One of these is the antimicrobial photodynamic therapy (aPDT), which is based on the combination of a so-called photosensitizer (PS), light of an appropriate wavelength and molecular oxygen (12, 13). Upon absorption of a photon, the PS molecule transfers from its ground state to an excited singlet state, wherefrom it can return to its ground state by two mechanisms generating reactive oxygen species (ROS), as follows: in type I mechanism, charge is transferred to a given substrate leading to formation of superoxide radicals (O^2−•^), hydrogen peroxide (H_2_O_2_), and free hydroxyl radicals (HO^•^). In type II mechanism, energy, but no charge is transferred directly to molecular oxygen (^3^O_2_), leading to the generation of singlet oxygen (^1^O_2_) (12, 13).

The antifungal efficacy of aPDT toward *C. albicans* was shown by many groups using different classes of PS (7, 14, 15). The porphyrin TMPyP was identified as effective PS for photokilling of *C. albicans* (7, 14). However, until now, evidence about the antifungal mechanism of action that leads to inactivation of *C. albicans* is scarce. Therefore, the aim of this study was to determine potential changes in the spatial distribution of the porphyrin PS TMPyP before and after irradiation with blue light.

## Materials and Methods

### Microorganism

*C. albicans* ATCC MYA-273 was obtained from the German Collection of Microorganisms and Cell Cultures (Deutsche Sammlung von Mikroorganismen und Zellkulturen, DSMZ; Braunschweig, Germany), was suspended in 5 mL Sabouraud dextrose broth (SDB) (Carl Roth, Karlsruhe, Germany) and incubated at 37°C overnight on a horizontal shaker device. When reaching the stationary phase of growth, the *C. albicans* suspension was centrifuged (Megafuge 1.0, Heraeus, Hanau, Germany) at 3.000 rpm and resuspended in 5 mL Millipore water. The number of *C. albicans* cells within the suspension was determined by manual enumeration using a chamber cell counter (Neubauer).

### Photosensitizer

TMPyP [5, 10, 15, 20-tetrakis(1-methylpyridinium-4-yl)-porphyrin tetra p-toluenesulfonate] was obtained from Sigma Aldrich (St. Louis, MO, USA). Its purity is specified to be 97%. 6,8 μg TMPyP were diluted in 5 mL Millipore water obtaining a stock concentration of 1 mM. Different concentrations of TMPyP were established by dilution with Millipore water. The TMPyP solutions were stored at 4°C in the dark.

### Light Source

The incoherent light source BlueV UV802L was provided by Waldmann Medizintechnik (Villingen-Schwenningen, Germany), its surface power density at the site of irradiation was ascertained as 20 mW/cm^2^. The UV802L shows continuous light emission from 380 to 470 nm with maximum at 420 nm and full width at half maximum (FWHM) of 30 nm, overlaid by two discrete narrow peaks at 405 and 436 nm.

### Phototoxicity Tests With *C. albicans*

100 μL of the *C. albicans* cell suspension and 100 μL PS of different concentrations (0–50 μM) were incubated for 15 min in a 96-well plate. The total volume of 200 μL was then irradiated with blue light for 10 min (20 mW/cm^2^; 12 J/cm^2^). The illumination was performed from the bottom side of the well-plate to avoid irregular refraction effects due to the uneven liquid surface. Controls were performed, as follows:

- Untreated control: Candida cells were not exposed to photosensitizer or light (P-, L-)
- Light control: Candida cells were irradiated only but no photosensitizer was added (P-, L+)
- Dark control: Candida cells were incubated with the photosensitizer, but were not irradiated (P+, L-)

Serial 10-fold dilutions with SDB (10^−1^ to10^−7^) were prepared from the irradiated samples and the controls. 60 μL of each dilution step were seeded on Sabouraud dextrose agar (SDA) using a modified method of Miles et al. (16), and incubated at 37°C in the dark. The number of colony forming units (CFU) was determined after 12 h of incubation.

### Analysis of the Phototoxicity Tests

All CFU results shown as medians, minima and maxima, were calculated using SPSS, v. 25 (SPSS Inc., Chicago, IL, United States) from the values of three independent experiments. The horizontal solid line in the graph represents reduction of 3 log_10_ steps of CFU, compared to the untreated control group. Medians on or below this line demonstrate an antifungal efficacy of 99.9% (3 log_10_), which is declared as biologically relevant antimicrobial activity (17, 18).

### Accumulation Measurement With *C. albicans*

750 μL of the *C. albicans* cell suspension and 750 μL PS (10 μM or 50 μM) were incubated in the dark for 15 min. The total volume of 1,5 mL was then centrifuged at 13.000 rpm (Centrifuge 5415R, Eppendorf, Hamburg, Germany) for 10 min. The supernatant was transferred to an acrylic cuvette to determine the absorbance (OD) of TMPyP using a spectral photometer (Specord 50 plus, Analytik Jena, Jena, Germany). The supernatant of the samples incubated with 50 μM were diluted in Millipore water using a dilution factor of 1:5 before spectral analysis. The resulting spectrum was compared to a spectrum of PS alone. The difference in the absorbance values points to the amount of PS, which has been resorbed or attached by *C. albicans*.

### Spatial Distribution of TMPyP in *C. albicans*

TMPyP emits fluorescence upon excitation, which allows the detection of TMPyP using fluorescence microscopy. For more accurate interpretation of the spatial distribution of TMPyP Wheat Germ Agglutinin was used for it is known to stain the cell wall of yeasts.

#### Incubation of *C. albicans* With TMPyP

250 μL of TMPyP and *C. albicans* suspension were incubated in absence of light for 15 min, yielding a TMPyP concentration of 1 μM. After incubation one sample was not treated further. A second sample was irradiated with the photodynamic light source described above (20 mW/cm^2^; 12 J/cm^2^) for 10 min in order to induce a photodynamic effect. Both samples were kept in the dark for further analysis with fluorescence microscopy.

#### Incubation of *C. albicans* With Wheat Germ Agglutinin

Wheat Germ Agglutinin Alexa Fluor® 488 conjugate (WGA, Life Technologies GmbH, Darmstadt, Germany) was used to label the cell wall of *C. albicans*. 50 μL WGA stock solution (1 mg/mL) and 450 μL *C. albicans* suspension in 0.25% Bovine-Serum-Albumin (BSA)–sodium-chloride (NaCl)-solution (Sigma Aldrich, Steinheim, Germany and Merck KGaA, Darmstadt, Germany, respectively) were incubated in the dark for 15 min. The samples were centrifuged for 10 min at 13.000 rpm (Centrifuge 5415R, Eppendorf, Hamburg, Germany) and the supernatant was discarded. The samples were then resuspended in BSA-NaCl-solution for further analysis with fluorescence microscopy.

#### Incubation of *C. albicans* With TMPyP and WGA

A *C. albicans* suspension in BSA-NaCl-solution was incubated with WGA stock solution for 15 min equivalent to 50 μg/mL WGA concentration. The samples were centrifuged for 10 min at 13.000 rpm and the supernatants were discarded. Then, identical volumes of TMPyP and Millipore water were added to the samples and were incubated for 15 min yielding a total TMPyP concentration of 1 μM. The samples were again centrifuged as described above, the supernatants were discarded and the samples were resuspended in Millipore water. Again, only one of the two samples was irradiated with the photodynamic light source, whereas the other one remained untreated for examining the localization of TMPyP and WGA before irradiation. Both samples were kept in the dark for further analysis with fluorescence microscopy.

#### Fluorescence Microscopy

A multichannel-3D-fluoresence-microscope (AxioImager Z1, Carl Zeiss AG, Oberkochen, Germany) comprising of six objectives (5x, 10x, 20x, 40x, 60x, and 100x magnification) was used in order to investigate the spatial distribution of the PS TMPyP. In this study, we used the fluorescence filters “RedFP” and “Oregon Green.” An overview of the excitation and emission wavelengths of the filters is shown in Table 1. Micrographs were taken with the AxioVision Rel. 4.9.1 software package (Zeiss, Oberkochen, Germany).

**Table 1:** Setup of the used fluorescence filters and their excitation and emission wavelengths, as well as the corresponding photosensitizers, that were examined by fluorescence microscopy.

**Filter**	**Target**	**Excitation (CWL/BW)**	**Emission (CWL/BW)**
RedFP	TMPyP	580/20	630/60
Oregon Green	WGA	500/20	535/30

*CWL, Central wavelength; BW, Bandwidth*.

## Results

### Phototoxicity Tests With *C. albicans*

**Figure 1:**
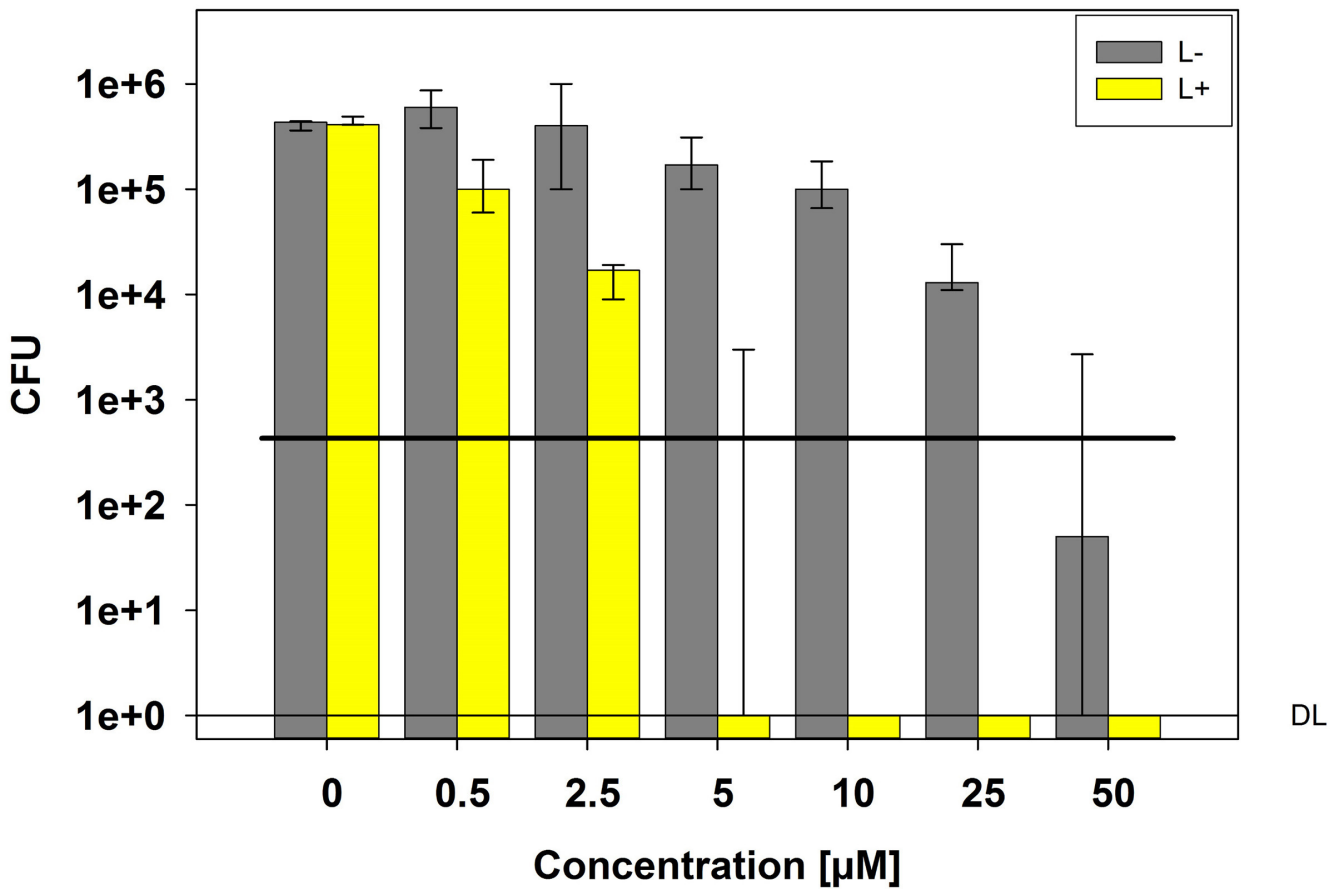
Phototoxicity test with *C. albicans* and TMPyP. *C. albicans* cells were incubated with different concentrations of TMPyP for 15 min. The samples were irradiated with blue light and an energy dose of 12 J/cm^2^ (L+). Non-irradiated controls (L-, gray bars) were conducted with *C. albicans* alone (0 μM) or with *C. albicans* and TMPyP (0,5–50 μM). CFU assays were performed after irradiation and CFU were determined after 12 h of incubation. Bars represent the medians, minima and maxima of three independent experiments (*n* = 3). The black line marks a CFU-reduction of 3 log_10_ (99.9%) as compared to the untreated control (0 μM TMPyP, L-). DL represents the detection limit.

Photokilling of *C. albicans* was achievable with a TMPyP concentration of 5 μM and higher, reaching a reduction in viability of 5 log_10_ CFU (99.999%) (Figure 1). Neither light irradiation alone nor incubation with TMPyP without light exposure caused a toxic effect on *C. albicans*. However, we could find a decrease in the viability of *C. albicans* without light exposure (dark control) from 25 μM on. This indicates, that concentrations of 25 μM TMPyP or above have a toxic effect on *C. albicans*.

### *C. albicans* TMPyP Accumulation

**Figure 2:**
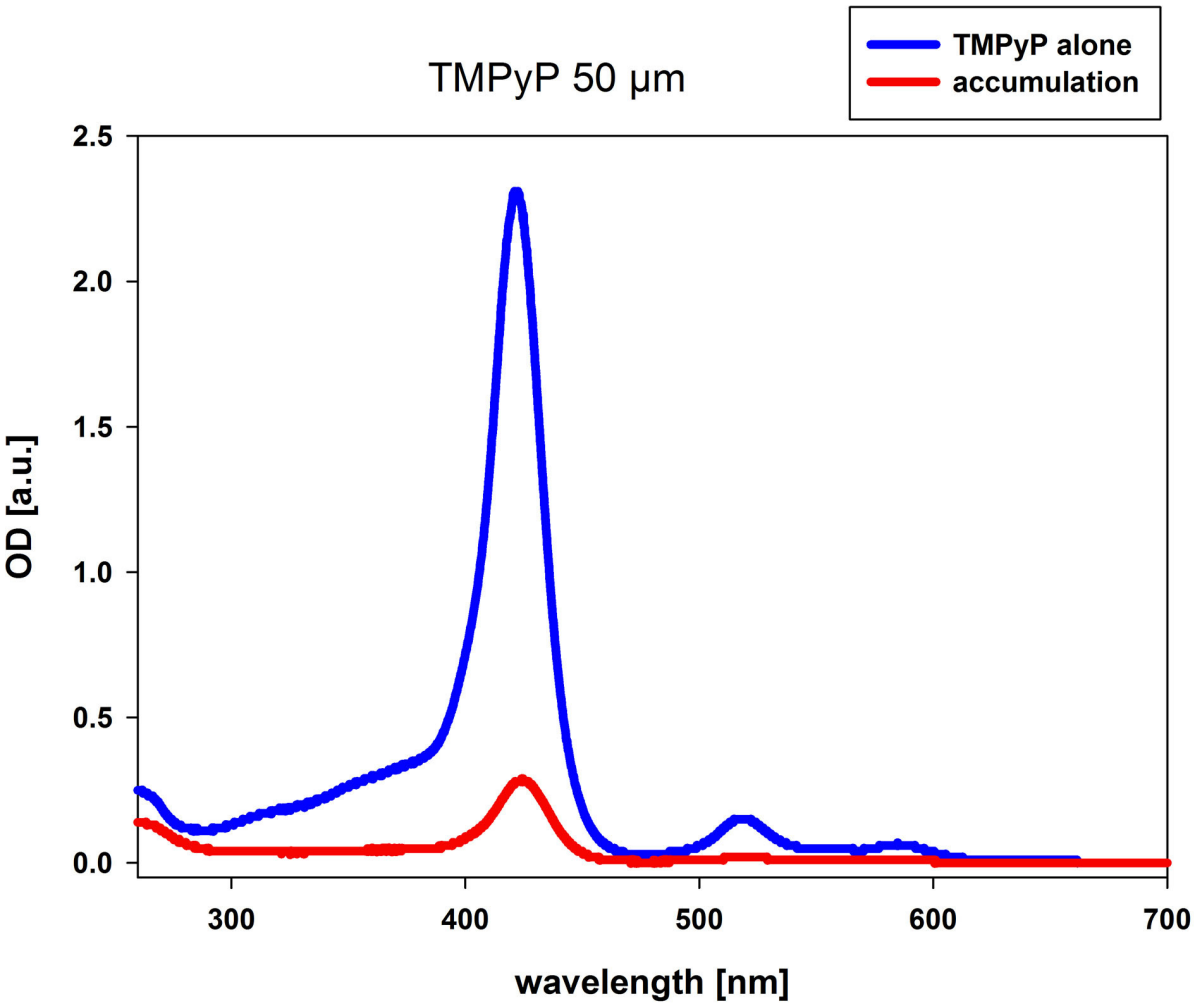
Accumulation of TMPyP in *C. albicans*. *C. albicans* was incubated with 50 μM TMPyP for 15 min in the dark. The sample was centrifuged and the OD of the supernatant was determined by using a spectral photometer. The supernatant of the sample was diluted in Millipore water using a dilution factor of 1:5 before spectral analysis. We compared the obtained spectrum (red line, accumulation) to the spectrum of TMPyP alone (blue line). The difference in the OD values refers to the amount of PS that has been resorbed or attached by *C. albicans*.

The accumulation measurement was performed with TMPyP concentrations of 10 and 50 μM, respectively. The difference in the absorbance values of PS alone and the suspension of *C. albicans* and PS after incubation refers to the amount of PS that has been resorbed by the fungal cells. At both concentrations (data of 10 μM not shown), we observed accumulation of TMPyP as indicated by the difference in the area under the curve or integrated optical density (Figure 2).

### Spatial Distribution of TMPyP in *C. albicans* Before Irradiation

**Figure 3:**
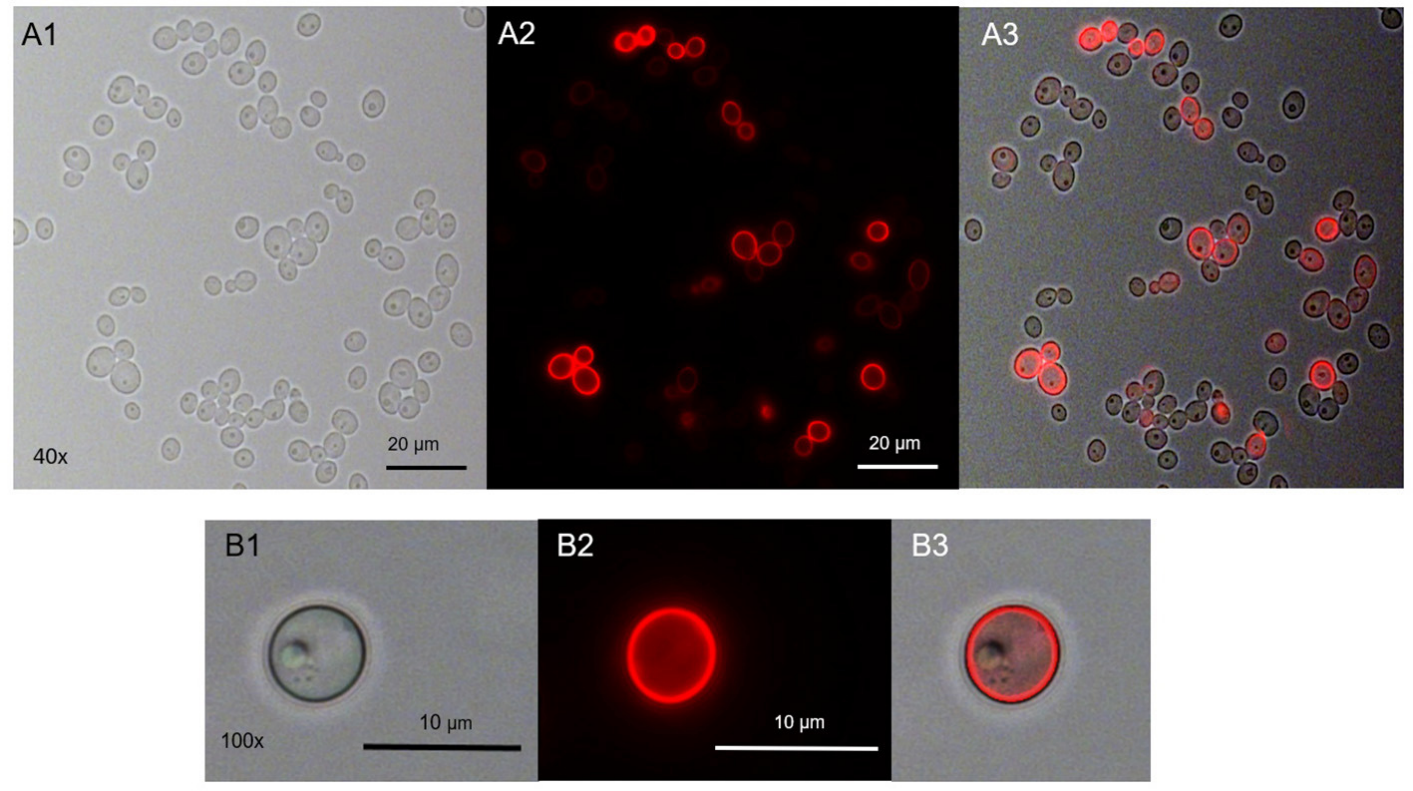
Spatial distribution of TMPyP in *C. albicans* before irradiation. *C. albicans* was incubated with 1 μM TMPyP for 15 min and kept in the dark. Fluorescence microscopy revealed a ring-shaped fluorescence pattern of TMPyP suggesting that TMPyP accumulates in the cells' periphery before irradiation. **A1** and **B1** show *C. albicans* cells incubated with TMPyP that were imaged by brightfield microscopy, **A2** and **B2** show the same cells imaged by fluorescence microscopy (filter: RedFP) and **A3** and **B3** show the overlay of brightfield and fluorescence micrographs.

Range finding experiments revealed that a concentration of 1 μM was suitable to visualize the localization of TMPyP in a ring-shaped configuration around the cells, but not in the cytoplasm of the cells (Figure 3). In addition, the number of fluorescent Candida cells and the total number of Candida cells (bright field image) of independent images were counted and evaluated. Table 2 shows the overall number of fluorescent cells compared to the total number of cells per image. The overall ratio of fluorescent Candida cells was 0.89.

**Table 2:** Quantification of Candida cells incubated with TMPyP.

Sample (image)^*^	**Number of cells in the brightfield**	**Number of cells fluorescing**	**Ratio**
1	109	53	0.49
2	66	60	0.90
3	23	23	1.00
4	103	101	0.98
5	21	21	1.00
6	49	48	0.98

* *Images of six independent experiments*.

### Verifying the Spatial Distribution of TMPyP With Wheat Germ Agglutinin

**Figure 4:**
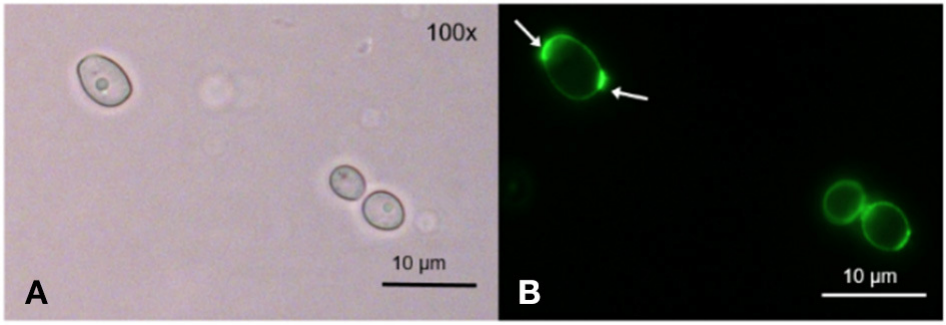
Spatial distribution of WGA in *C. albicans*. *C. albicans* was incubated with 100 μg/mL WGA to stain the yeast's cell wall. WGA is marking the cell wall homogeneously at low intensity and certain areas at high intensity, most likely bud scars (shown here) and cell buds ( → ). **(A)** shows *C. albicans* cells incubated with WGA and imaged by brightfield microscopy and **(B)** shows the same cells imaged by fluorescence microscopy (filter: Oregon Green).

After incubation with TMPyP, we stained the fungal cells with Wheat Germ Agglutinin (WGA). The incubation of *C. albicans* with WGA also resulted in a ring-shaped arrangement, however, in contrast to TMPyP the distribution of WGA was not homogeneous. Although the cell wall was homogeneously stained, we found strong accumulation in areas close to the cell wall that correspond well with cell buds and bud scars (Figure 4).

**Figure 5:**
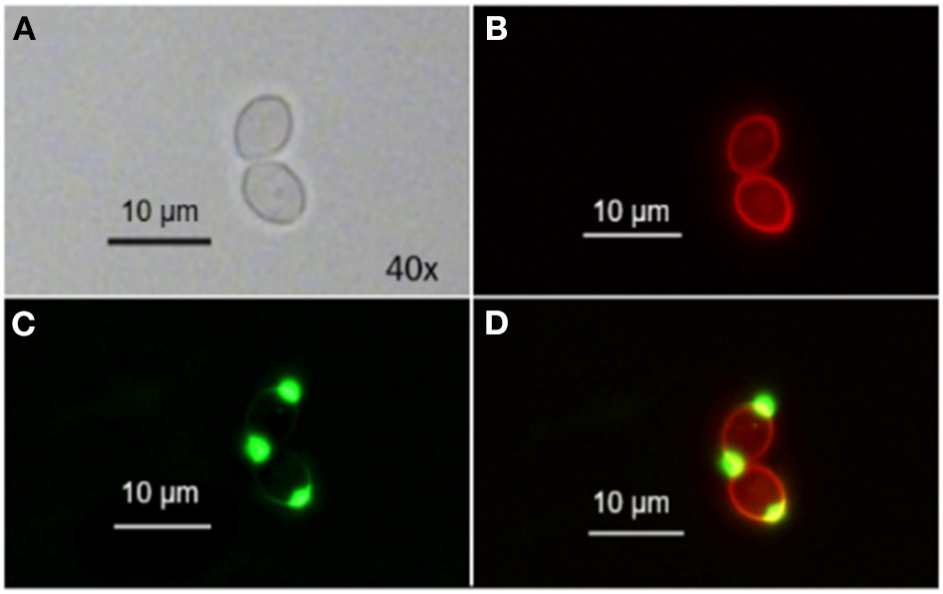
Spatial distribution of WGA and TMPyP in *C. albicans* before irradiation. *C. albicans* cells were incubated with 50 μM WGA and 1 μM TMPyP for 15 min and kept in the dark. Using fluorescence microscopy, we observed colocalization of fluorescence signals from WGA and TMPyP within the cell wall, but bud scars were not marked by TMPyP. **(A)** shows *C. albicans* cells incubated with WGA and TMPyP imaged by brightfield microscopy, **(B)** shows fluorescence signals of TMPyP within the same cells (filter: RedFP) imaged by fluorescence microscopy, **(C)** shows fluorescence signals of WGA within the same cells (filter: Oregon Green) imaged by fluorescence microscopy and **(D)** shows the overlay of both fluorescence signals.

When co-incubating *C. albicans* with TMPyP and WGA we could observe a red fluorescing ring generated by the porphyrin that was congruent with green fluorescing signals from WGA in the cell wall, while cell buds and bud scars were not stained by TMPyP (Figure 5).

### Localization of TMPyP After Irradiation

After incubation of *C. albicans* with TMPyP we irradiated the sample with blue light for photodynamic action and subsequently examined the localization of the PS by fluorescence microscopy.

**Figure 6:**
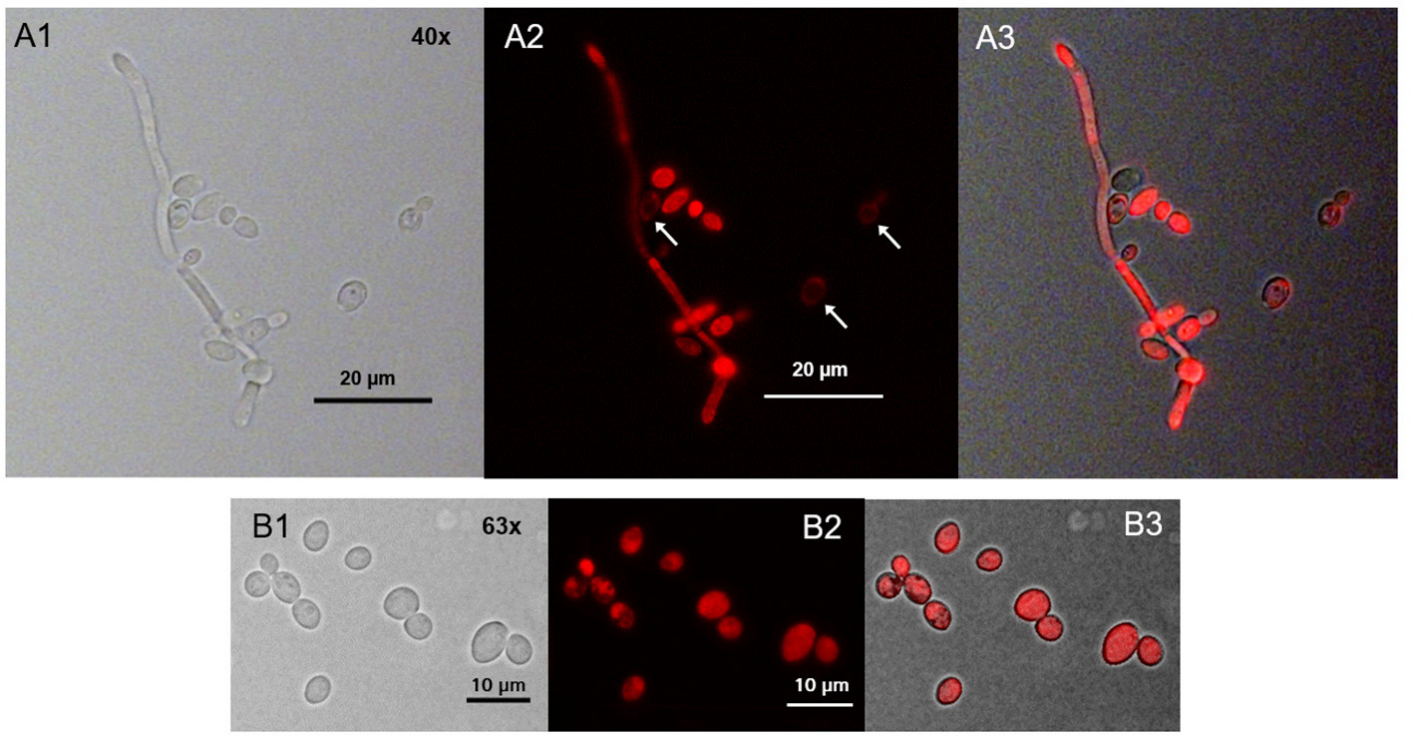
Spatial distribution of TMPyP in *C. albicans* after irradiation. *C. albicans* cells incubated with TMPyP (1 μM) were irradiated with blue light (12 J/cm^3^). Fluorescence microscopy revealed some ring-shaped configurations of TMPyP corresponding to the cell wall (**A2**, → ). Most of the cells showed TMPyP-fluorescence from the area of the cytoplasm. This indicates that TMPyP delocalizes from the cells' periphery to the cells' interior upon photodynamic action. *C. albicans* cells incubated with TMPyP imaged with brightfield microscopy **(A1, B1)**, fluorescence microscopy **(A2, B2)**, and brightfield and fluorescence micrographs overlayed **(A3, B3)**.

**Figure 7:**
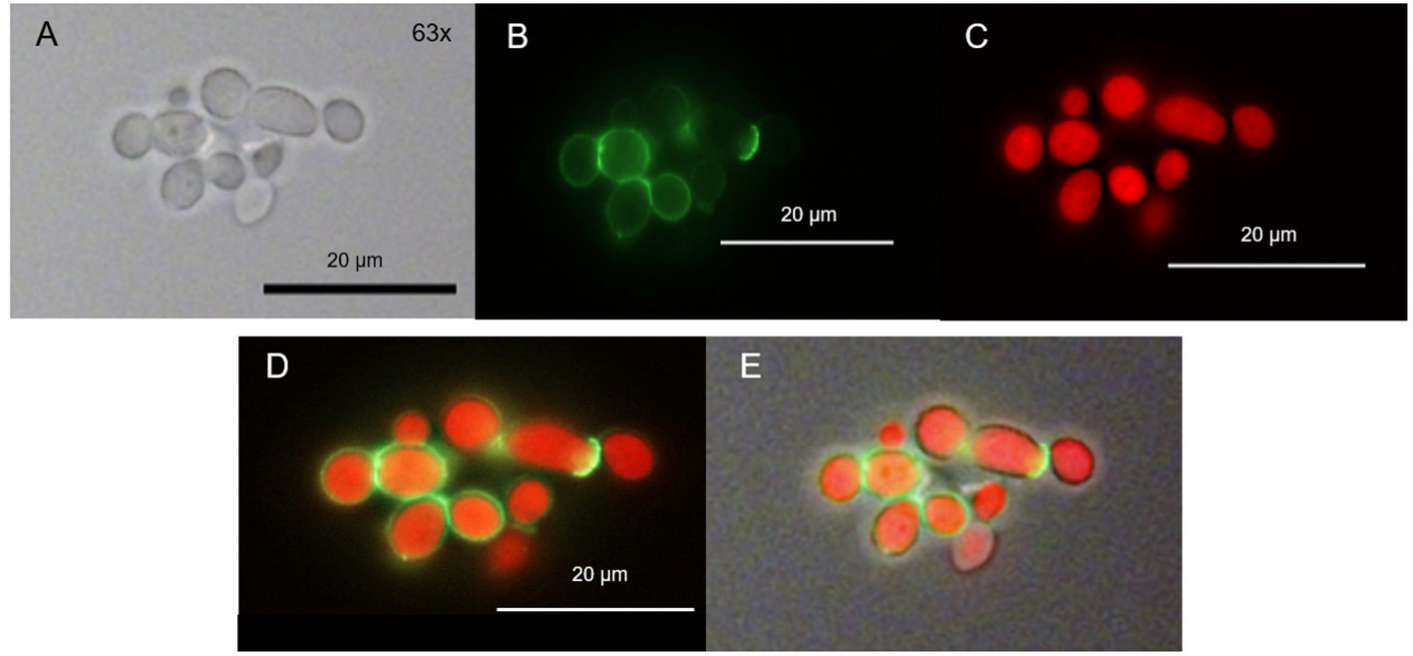
Spatial distribution of TMPyP and WGA in *C. albicans* after irradiation. *C. albicans* was incubated with TMPyP (1 μM) and WGA (50 μg/ml) and irradiated with blue light (12 J/cm^2^). With fluorescence microscopy WGA (green signal) accumulated in the area of the cell wall **(B,D,E)**. TMPyP-fluorescence (red signal) indicates homogenous distribution into the cytoplasm. *C. albicans* cells incubated with WGA and TMPyP imaged by brightfield microscopy **(A)**, fluorescence from WGA within the same cells (**B**, filter: Oregon Green, fluorescence microscopy), fluorescence signals of TMPyP within the same cells (**C**, filter: RedFP, fluorescence microscopy), overlay of both fluorescence signals **(D)** and overlay of the fluorescence signals and the brightfield micrograph **(E)**.

We found that only few ring-shaped fluorescence patterns remained. Most of the cells, however, showed a homogenous TMPyP distribution across the cytoplasm. TMPyP seems to delocalize upon illumination and enters the cytosol of *C. albicans* (Figure 6). When incubating *C. albicans* with TMPyP and WGA, WGA remained bound to the cell wall while TMPyP seemed to have entered the interior of the cells (Figure 7).

## Discussion

In this study, we examined the mechanism of action that leads to inactivation of *C. albicans* in the course of aPDT with TMPyP, a four-fould positively charged porphyrin derivate. The ^1^O_2_ quantum yield Φ_Δ_ of TMPyP, which means the amount of type II reaction, was specified to be 0,74 (19). Thus, the photoactivation of TMPyP results in the generation of a substantial amount of singlet oxygen. TMPyP has been used in numerous studies before, mainly describing its effect on bacteria (20–25). In the present study, a CFU reduction of 5 log_10_ steps of *C. albicans* was obtained by using a concentration of 5 μM TMPyP and an energy dose of 12 J/cm^2^. Other groups also demonstrated a reduction up to 5 log_10_ employing the same PS concentrations but longer incubation and irradiation periods (14, 26). A photodynamic antifungal effect on *C. albicans* could even be demonstrated by incubating the yeasts with a TMPyP concentration of 2 μM for 15 min and applying an energy dose of 12.1 J/cm^2^ (24). Smaller differences between studies may be attributed to use of light sources with different spectra. Nevertheless, there is evidence, that TMPyP has a toxic effect on *C. albicans* also without light exposure. Quiroga et al. observed a decrease of 1.5 log_10_ in the viability of *C. albicans* in the dark control samples using a PS concentration of 10 μM (14). This matches the results in our study, but we found a toxic effect of TMPyP only at a concentration of 25 μM or higher. Compared to most bacteria, photokilling of fungal cells needs higher PS concentrations to achieve similar inactivation rates (6, 7). For instance, methicillin-resistant *Staphylococcus aureus* (MRSA) and enterohemorrhagic *Escherichia coli* (EHEC) can be easily reduced by more than 3 log_10_ steps using 0.5 μM or 1 μM TMPyP, respectively and an energy dose of 0.5 J/cm^2^ (7), while in our study a TMPyP concentration of 5 μM and an energy dose of 12 J/cm^2^ were necessary for photokilling of *C. albicans*. The higher tolerance of fungal cells against aPDT may be due to their different cell structure (7). Fungi have a nuclear membrane, which forms a barrier to penetration of the PS itself as well as of ROS (6, 27). Furthermore, fungal cells are larger than bacterial cells, thus needing a bigger amount of ROS to be inactivated (6). Accumulation measurements showed that TMPyP is absorbed by *C. albicans*, which is an essential requirement for the photodynamic effect. The accumulation of TMPyP is known from former studies, where a high affinity of porphyrins to *C. albicans* was shown (14, 26, 28).

Using fluorescence microscopy, we found that before irradiation TMPyP localizes in a ring-shaped fluorescence pattern around the *C. albicans* cells, but not inside the yeast cells, i.e., cytoplasm or cytosol. Therefore, we assumed the cell wall to be the primary binding site for TMPyP. Likewise, Smijs et al. concluded in their study that Sylsens B, a porphyrin derivate, localizes within the cell wall of *Trichophyton rubrum* (29). To confirm this assumption, we stained *C. albicans* with WGA as well as with WGA and TMPyP. WGA specifically binds to N-acetyl-D-glucosamine, which is used by *Candida* to synthesize chitin, an essential part of the fungal cell wall (30). Therefore, WGA can be used as a marker for the fungal cell wall. We found that the distribution of WGA was not homogenous. There were parts of the cell wall which contained more WGA than others. From what is known about yeast cell morphology, these structures are corresponding well with cell buds and bud scars, which are remnants of the septum that is formed between mother and daughter cells during mitosis. These bud scars on the cell surface containhigh concentrations of chitin and consequently WGA as well (5, 31). The buds themselves also contain ample amounts of N-acetyl-D-glucosamine, because they are highly actively synthesizing chitin during growth. Co-incubation with TMPyP and WGA showed that the structures showing fluorescence caused by the porphyrin and the cell wall marked with green fluorescence from WGA were congruent. In contrast to WGA, TMPyP is not covalently bound to components of the fungal cell wall, which may explain why TMPyP does not concentrate in bud scars, but leads to a homogenous fluorescence signal around the cells. However, co-incubation with TMPyP and WGA showed that the structures showing fluorescence caused by the porphyrin and the cell wall marked with green fluorescence from WGA were congruent. This means that both dyes localize at the same place, which is the fungal cell wall. After irradiation with blue light the spatial distribution of TMPyP changed. Although a ring-shaped pattern could be observed occasionally, most yeast cells showed fluorescence across the whole cell. This may be an indication that upon irradiation the cell membrane becomes permeable and the PS can enter the cytosol inside the cell, which finally leads to cell death. Lambrechts et al. conducted similar studies using the cationic porphyrin derivate TriP[4] (32). In contrast to TMPyP, TriP[4] has three positive charges. Using confocal fluorescence microscopy and a PS concentration of 25 μM they could also show ring shaped fluorescence patterns before illumination and supposed that TriP[4] binds to the cell wall of *C. albicans* (32). By staining the cell wall with WGA, we proved that this assumption is also true for TMPyP. After irradiation, Lambrechts et al. described a massive influx of TriP[4] due to a damage of the yeasts' cell membrane. This lead to fluorescence patterns coming from the whole cell despite a small organelle, which they identified as the cell vacuole (32). In our study, we could also show the fluorescence of the whole cell after irradiation, whereas WGA remained bound to the cell wall. This can be explained by the reversible bond of TMPyP to the cell wall which is not as strong as the covalent bond of WGA to N-acetyl-D-glucosamine that is used for chitin synthesis (14, 30). Thus, we presume that ROS produced during the aPDT process attack the cell membrane, leading to an irreversible damage that causes the fungal cells to die. Due to the reversible bond of TMPyP to the cell wall, it can penetrate flaws, voids and clefts of the cell membrane and can finally enter the cytosol. Bertoloni et al. studied the photodynamic effect of hematoporphyrin on *C. albicans*. Electron-microscopic pictures revealed a change in the structure of the cell wall after irradiation. The cells became less electron-dense and the cell wall took on a more and more jagged form (33). Liu et al. showed the loss of intracellular proteins due to a damage of the cell membrane by using 10 μM hematoporphyrin monomethyl ether and an energy dose of 72 J/cm^2^ for the aPDT against *C. albicans* (34), supporting our assumption that the cell membrane is the target structure to be disrupted due to photodynamic action in presence of TMPyP.

Increasing resistance against common antifungal medication such as azoles and echinocandins is a continuously growing challenge for the world's health systems (35, 36). Resistance mechanisms leading to an acquired resistance to antifungal drugs include an overexpression of the drug target (37, 38) as well as an upregulation of efflux pumps (2, 39). In contrast to common antifungal agents, it is very unlikely that aPDT can lead to a resistance because the photodynamic approach is an unselective treatment, which focuses on a variety of targets (12, 13). Efflux pumps must be considered to have a negative influence on photodynamic inactivation. However, among the common classes of PS only phenothiazinium dyes act as a substrate for efflux pumps (40). Furthermore, we could show that the internalization of TMPyP is not a requirement for the effectiveness of the photodynamic approach, but its result. By using fluorescence microscopy, it became evident that before irradiation, TMPyP accumulates within the cell wall, while its target structure seems to be the cell membrane, and it does not enter the cytosol before the photodynamic action is triggered. Due to this fact, there is no interaction between TMPyP and fungal DNA, which also reduces the likeliness for the development of resistances.

The aPDT therefore seems to be a promising tool in the control of microorganisms which show resistances against common antimicrobial treatments. Schafer et al. showed that even *Deinococcus radiodurans*, which is considered as one of the toughest bacteria in the world, can be killed quickly and successfully using the photodynamic principle (41). Therefore, it is likely that this new antimicrobial photodynamic principle has realistic chances of being used in the medical field in the future.

## Conclusion

Medical care is confronted with an increase of serious infections attributed to multi-resistant fungi during the last years and is in need for alternative therapies. Antimicrobial photodynamic therapy is a promising approach in this field. We could show that an inactivation of *C. albicans* can be achieved using TMPyP, a 4-fold positively charged porphyrin derivative. The examination of the spatial distribution of the PS by using fluorescence microscopy revealed an accumulation of TMPyP in the cell wall, when the yeasts were kept in the dark. Upon irradiation the PS migrates to the cytosol. This suggests that the photodynamic action might be mainly due to damage of the cell membrane, which becomes permeable for the porphyrin-based PS TMPyP. Due to the fact that the mechanism of action seems to be located in the cell wall and cell membrane, but not in the cytoplasm, it is unlikely that *C. albicans* can develop resistances against the photodynamic process. These findings may contribute to further development and modifications of photosensitizers to make them more effective in their use against microbial infections.

## Data Availability Statement

The original contributions generated for this study are included in the article/supplementary material, further inquiries can be directed to the corresponding author/s.

## Author Contributions

TV: conceived and designed the experiments, performed the experiments, analyzed the data, and wrote the manuscript with input from all authors. TM and AG: conceived and designed the experiments and analyzed the data. FC and WB: conceived and designed the experiments. JR and K-AH: analyzed the data. All authors contributed to the article and approved the submitted version.

## Conflict of Interest

The authors declare that the research was conducted in the absence of any commercial or financial relationships that could be construed as a potential conflict of interest.
